# Quantification of microcystin production and biodegradation rates in the western basin of Lake Erie

**DOI:** 10.1002/lno.12096

**Published:** 2022-05-04

**Authors:** Justin D. Chaffin, Judy A. Westrick, Elliot Furr, Johnna A. Birbeck, Laura A. Reitz, Keara Stanislawczyk, Wei Li, Peter K. Weber, Thomas B. Bridgeman, Timothy W. Davis, Xavier Mayali

**Affiliations:** ^1^ F.T. Stone Laboratory and Ohio Sea Grant The Ohio State University Put‐In‐Bay Ohio USA; ^2^ Lumigen Instrument Center Wayne State University Detroit Michigan USA; ^3^ Department of Biological Sciences Bowling Green State University Bowling Green Ohio USA; ^4^ Physical and Life Sciences Directorate Lawrence Livermore National Laboratory Livermore California USA; ^5^ Lake Erie Center University of Toledo Oregon Ohio USA; ^6^ Present address: Department of Earth and Environmental Sciences University of Michigan Ann Arbor Michigan USA

## Abstract

Cyanobacterial biomass forecasts currently cannot predict the concentrations of microcystin, one of the most ubiquitous cyanotoxins that threaten human and wildlife health globally. Mechanistic insights into how microcystin production and biodegradation by heterotrophic bacteria change spatially and throughout the bloom season can aid in toxin concentration forecasts. We quantified microcystin production and biodegradation during two growth seasons in two western Lake Erie sites with different physicochemical properties commonly plagued by summer *Microcystis* blooms. Microcystin production rates were greater with elevated nutrients than under ambient conditions and were highest nearshore during the initial phases of the bloom, and production rates were lower in later bloom phases. We examined biodegradation rates of the most common and toxic microcystin by adding extracellular stable isotope‐labeled microcystin‐LR (1 *μ*g L^−1^), which remained stable in the abiotic treatment (without bacteria) with minimal adsorption onto sediment, but strongly decreased in all unaltered biotic treatments, suggesting biodegradation. Greatest biodegradation rates (highest of −8.76 d^−1^, equivalent to the removal of 99.98% in 18 h) were observed during peak bloom conditions, while lower rates were observed with lower cyanobacteria biomass. Cell‐specific nitrogen incorporation from microcystin‐LR by nanoscale imaging mass spectrometry showed that a small percentage of the heterotrophic bacterial community actively degraded microcystin‐LR. Microcystin production and biodegradation rates, combined with the microcystin incorporation by single cells, suggest that microcystin predictive models could be improved by incorporating toxin production and biodegradation rates, which are influenced by cyanobacterial bloom stage (early vs. late bloom), nutrient availability, and bacterial community composition.

Cyanobacterial harmful algal blooms (HABs) are globally distributed due to excess loading of nutrients (nitrogen [N] and phosphorus [P]) and elevated temperatures associated with climate change (Paerl and Huisman 2008; O'Neil et al. 2012). Cyanobacterial HABs pose threats to human health through the production of toxic secondary metabolites called cyanotoxins (Carmichael 1992). The high biomass of cyanobacterial blooms also disturb food web structure (Tillmanns et al. 2008) and can create hypoxic conditions when degraded (Watson et al. 2016). Microcystins are the most common and diverse cyanotoxins with over 200 known congeners, and microcystin‐LR (named for the two amino acid side groups leucine [L] and arginine [R]) is one of the most common and toxic congeners (Harke et al. 2016; Spoof and Catherine 2017). In fact, to date, the presence of microcystins and the cosmopolitan microcystin‐producer *Microcystis* have been confirmed in 79 countries worldwide (Harke et al. 2016). Since the 1990s, there have been periodic “do not use tap water” advisories issued for many countries worldwide due to elevated microcystin concentrations (Pouria et al. 1998; Qin et al. 2010; Sitoki et al. 2012; Steffen et al. 2017). Understanding the environmental drivers that affect microcystin concentrations and developing forecasting capabilities is paramount to protect human and environmental health and ultimately prevent future cyanobacterial HABs.

Cyanobacterial biomass forecasts in Lake Erie, a mesotrophic lake with a history of cyanobacterial HABs, are made possible with frequent (near‐daily) remote sensing data (Wynne et al. 2013) and lake water mass movement simulations (Rowe et al. 2016; Xue et al. 2016). One of the more widely known forecasts is from the National Aeronautics and Space Administration and National Oceanic and Atmospheric Administration that utilizes satellite images of Lake Erie and incorporates cyanobacterial biomass into the Finite Volume Community Ocean Model to forecast cyanobacteria location and biomass 2–4 d in advance. While the biomass forecasts have aided managers in predicting when and where the cyanobacterial blooms will be located, the forecasts cannot currently predict microcystin concentrations. Remote sensing cannot detect microcystins, and there is no consistent biomass‐to‐microcystin ratio (Stumpf et al. 2016). Insights on how microcystin production changes spatially and temporally in a bloom could help overcome the limitations that currently prevent microcystin concentration forecasts.

Forecasting microcystins requires not only understanding microcystin production but also microcystin biodegradation. Little is known regarding microcystin biodegradation compared to production, particularly in natural systems. The cyclic microcystin structure makes these toxins very stable under natural solar irradiance, temperatures, and pH (Lahti et al. 1997; Wörmer et al. 2010). Microcystins can be removed from the water column via biodegradation by heterotrophic bacteria (Jones et al. 1994; Cousins et al. 1996) and binding to suspended clays (Morris et al. 2000). In order for biodegradation to occur, the cyanobacterial cell must first be lysed, which occurs naturally during programmed cell death (Ross et al. 2006), zooplankton grazing (Mohamed and Al‐Shehri 2013), chytrid infection and/or cyanophage attack (Steffen et al. 2017; McKindles et al. 2020). A study with Lake Erie water showed that 15 *μ*g L^−1^ dissolved microcystin spiked into a microcosm was completely lost (below detectable levels) within 24 h, whereas the abiotic treatment maintained stable microcystin concentrations, further suggesting abiotic factors (such as UV radiation) do not degrade microcystins (Mou et al. 2013). Another culture‐based experiment with Lake Erie water showed that a diverse community of heterotrophic bacteria from several phyla was needed to degrade microcystins (Thees et al. 2019).

The objective of this research was to experimentally derive naturally occurring microcystin production and biodegradation rates for the western basin of Lake Erie. For production rate experiments, ambient nutrient controls were used to estimate in situ production rates and P and N amendments were used to estimate rates of maximum potential production. For microcystin biodegradation, biotic and abiotic treatments were incubated with spikes of stable isotope (^15^N) labeled microcystin‐LR, which allow measurements distinct from the naturally occurring microcystin that is not labeled with ^15^N. Because the microcystin‐to‐cyanobacteria biomass ratio in western Lake Erie tends to decrease throughout the season (Gobler et al. 2016), we hypothesized that microcystin production rates would be greatest during the early stages of the bloom and that microcystin biodegradation rates would be highest at the end of the bloom.

## Materials and methods

### Study location and field methods

**Fig. 1:**
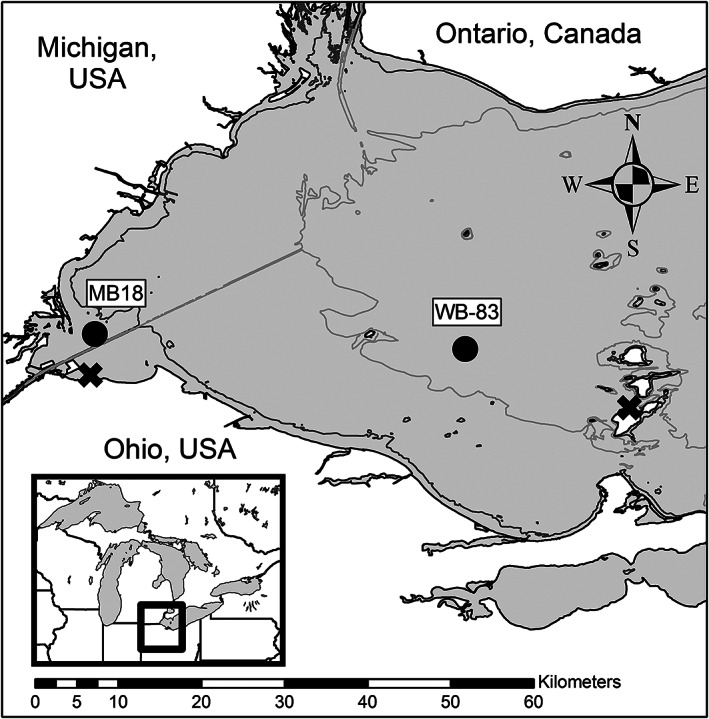
Sample locations (circles) used to collect water for microcystin production and biodegradation experiments and the incubation sites (X). Contour lines are 3 and 8 m in depth.

Two sample locations in western Lake Erie, which are part of long‐term monitoring programs, were selected to represent different water quality (Fig. 1). Site MB18 (depth ~ 2.5 m) is located in Maumee Bay and is heavily impacted by the nutrient‐rich discharge from the Maumee River (Bridgeman et al. 2012). Site WB‐83 (depth ~ 10 m) is located in the center of the western basin and has lower nutrient concentrations and cyanobacterial biomass due to decreased influence of the Maumee River (Bridgeman et al. 2012; Chaffin et al. 2013). Microscopy confirmed that *Microcystis* dominated the cyanobacterial communities at both sites during the sampling periods.

The two sites were sampled weekly to biweekly throughout the 2018 and 2019 HAB seasons (June to early October) to monitor concentrations of nutrients, physicochemical parameters (pH, conductivity, temperature, dissolved oxygen, turbidity), cyanobacterial‐chlorophyll *a* (Chl *a*) concentration as a surrogate for biomass, and total and extracellular microcystins concentrations. Field and sample analysis methods followed previous reports (Bridgeman et al. 2012; Chaffin et al. 2013). Microcystin data reported in this manuscript were analyzed by liquid chromatography with tandem mass spectrometry (LC–MS/MS; Birbeck et al. 2019), as described below. When microcystin production and biodegradation experiments were conducted, 40 L of surface water containing the natural assemblage of plankton was collected (*see* Supporting Information Material S1) and kept dark while in route back to the laboratory. The experiments began within 8 h after water collection.

### Microcystin production

Microcystin production experiments occurred biweekly at both sites July–October in 2018 and 2019 (10 experiments in both years). Twelve 2.5‐L clear polyethylene terephthalate glycol (PETG) bottles were prerinsed with 10% vol/vol hydrochloric acid (HCl) then rinsed three times with deionized (DI) water, followed by three more rinses with the collected lake water. The PETG bottles were filled from the two carboys (half from each carboy), and the carboys were inverted vigorously three times after every third bottle was filled. This ensured the water was well‐mixed and consistent among bottles. Three bottles served as the ambient nutrient level controls. One micromole per liter phosphate (as KH_2_PO_4_) was added to the nine amended bottles. To test how different forms of N affected microcystin production rate, 100 *μ*mol L^−1^ nitrate (NaNO_3_), ammonium (NH_4_Cl), or urea‐N (50 *μ*mol L^−1^ urea) were added alongside P, each in three replicates. We were not concerned with which nutrient (P or N) primarily limited growth and microcystin production. Bottles were incubated in situ in Lake Erie using a limnocorral covered by a screen that reduced sunlight by ~ 65% to prevent photoinhibition. The incubation site for the MB18 experiments was at the Lake Erie Center, whereas the incubation site for the WB‐83 experiments was at the Stone Laboratory (Fig. 1). The water temperature of the incubation sites was similar to those of the respective collection sites. Initial samples were collected from the carboys prior to incubation, and samples were collected every 24 h throughout the incubation, which lasted for 72 h.

At each sample time point, all bottles were removed from the limnocorrals. The bottles were then wiped clean to prevent external colonization by algae and biofilms and then were brought into the laboratory for processing and filtering. Each bottle was vigorously inverted several times prior to each sample aliquot poured. Bottles were placed back into the limnocorrals after samples were collected.

Phytoplankton community composition and cyanobacterial biomass were quantified immediately using a FluoroProbe (BBE moldaenke) equipped with a bench‐top cuvette reader, as previously described (Chaffin et al. 2013) (Supporting Information Material S1). The FluoroProbe cyanobacteria‐Chl *a* concentration data was used to calculate *Microcystis*‐dominated cyanobacteria growth rates.

Intracellular microcystins samples were collected by filtering 50–100 mL of whole water onto 1.2 *μ*m polycarbonate filters using a vacuum pump filtration manifold at 5 in Hg pressure. The filters were individually stored in amber glass vials with 5–10 mL of DI water for resuspension during extraction and frozen at −20°C until analysis. Microcystins were extracted using Ohio EPA's standardized three freeze/thaw cycle protocol to lyse cells (Ohio EPA 2018a). The cell lysate was filtered through a GMF filter (0.45 *μ*m) to remove cellular debris (Ohio EPA 2018a) into an amber glass vial and frozen at −20°C until analysis. LC–MS/MS was used to quantify total microcystins (Supporting Information Material S1).

DNA was collected by filtering 20–50 mL of sample water onto a 1.2 *μ*m polycarbonate filter and frozen at −80°C until extraction and analysis. After DNA extraction and DNA concentration and quality were measured (Supporting Information Material S1), qPCR quantification of the *mcyE* gene, 1 of the 10 genes in the *mcy* operon responsible for microcystin biosynthesis, was conducted with the Phytoxigene CyanoDTec multiplex assay (Diagnostic Technology). The Ohio EPA uses this protocol for the routine monitoring of cyanotoxins in source water (Ohio EPA 2018b, Supporting Information Material S1).

### Microcystin biodegradation and incorporation experiment

Microcystin biodegradation experiments were conducted four times at each site August–October 2019. One additional experiment was conducted during 2020 with MB18 water to quantify bacterial incorporation of microcystins. The microcystin biodegradation experiments consisted of two treatments, a biotic treatment and an abiotic control. Six 2.5 L PETG bottles (treatments in triplicate) were prerinsed with 10% vol/vol HCl then rinsed three times with DI water. For the biotic treatment, the three bottles were filled with lake water, whereas the abiotic control was filled with 0.22 *μ*m filtered lake water. All six bottles were spiked with ^15^N‐microcystin‐LR (*see* Supporting Information Material S1) to a final concentration of 1 *μ*g L^−1^, the World Health Organization guideline for microcystins in drinking water. Bottles were incubated in temperature and light‐controlled incubators at lake temperature recorded at time of collection and under a light intensity of 300 *μ*mol photons m^−2^ s^−1^ at a light: dark cycle to align with local sunrise and sunset time. Samples were collected every 3 h starting 3 h after the ^15^N‐microcystin‐LR spike for the first 24 h, then at Hours 36 and 48 to measure microcystins by LC–MS/MS.

Ten to twenty‐five milliliters samples, depending on biomass in the biotic treatment, were filtered through 0.4 *μ*m pore size polycarbonate filters (Whatman Nuclepore; GE Healthcare Life Sciences) and the filtrate was collected into a 40 mL amber glass vial. The filtrate and filter with plankton were stored at −80°C for later analysis of extracellular and intracellular ^15^N‐microcystin‐LR and ambient microcystins by LC–MS/MS (Supporting Information Material S1).

To quantify bacterial incorporation of ^15^N‐microcystin‐LR from the 2020 incubation, we collected cells from the degradation experiment at time points, 0, 2, 8, 24, and 48 h. Isotope imaging was performed with a CAMECA nanoscale imaging mass spectrometry (NanoSIMS) 50 at Lawrence Livermore National Laboratory (Samo et al. 2018). The fraction of cellular N incorporated from the substrate (N_net_) was calculated based on the initial control and the ^15^N enrichment of the ^15^N‐microcystin‐LR (Pett‐Ridge and Weber 2022) (*see* Supporting Information Material S1 for details).

### Data analysis

*Microcystis*‐dominated cyanobacteria and toxic strain growth was calculated using the equation: *μ* = (ln([*C*]_*t*_ − ln[*C*]_0_)/(*t*_*t*_ − *t*_0_), where *μ* is the specific growth rate, *C* is the concentration of cyanobacteria‐Chl *a* or *mcyE* gene copies at time (*t*) *t* and time 0.

Apparent first‐order kinetics was applied to determine rate constants for microcystin production and biodegradation by plotting the natural log of microcystin concentration vs. time and using a best fit line (Szlag et al. 2019). A positive rate indicated a net production, whereas a negative value indicated a net loss. Microcystin production in the biodegradation experiments (and vice versa) would complicate the rate calculations, and hence, our reason for measuring intracellular microcystins and ^15^N‐microcystin‐LR in the production and biodegradation experiments, respectively. Therefore, we do not have bulk measurements of community turnover.

A Shapiro–Wilk test of normality was conducted to confirm normally distributed data for growth, microcystin production and loss. Then a three‐way analysis of variances (ANOVA) was conducted to determine if there were significant differences among year, sample site, and treatment. Tukey tests were conducted to determine differences among nutrient treatments. Pearson correlations were determined for *Microcystis*‐dominated cyanobacteria growth, microcystin‐toxigenic cyanobacteria growth, and microcystin production rate constants. These statistical analyses were performed using IBM SPSS version 27.

A pairwise Wilcoxon rank‐sum test was carried out on the N isotope data (N_net_, or percentage of new biomass from ^15^N_10_‐microcystin‐LR) for *Microcystis* and non‐*Microcystis* cells collected from the 2020 experiment in R version 4.0.2 (R Core Team, 2017).

## Results

### Seasonal characteristics

**Fig. 2:**
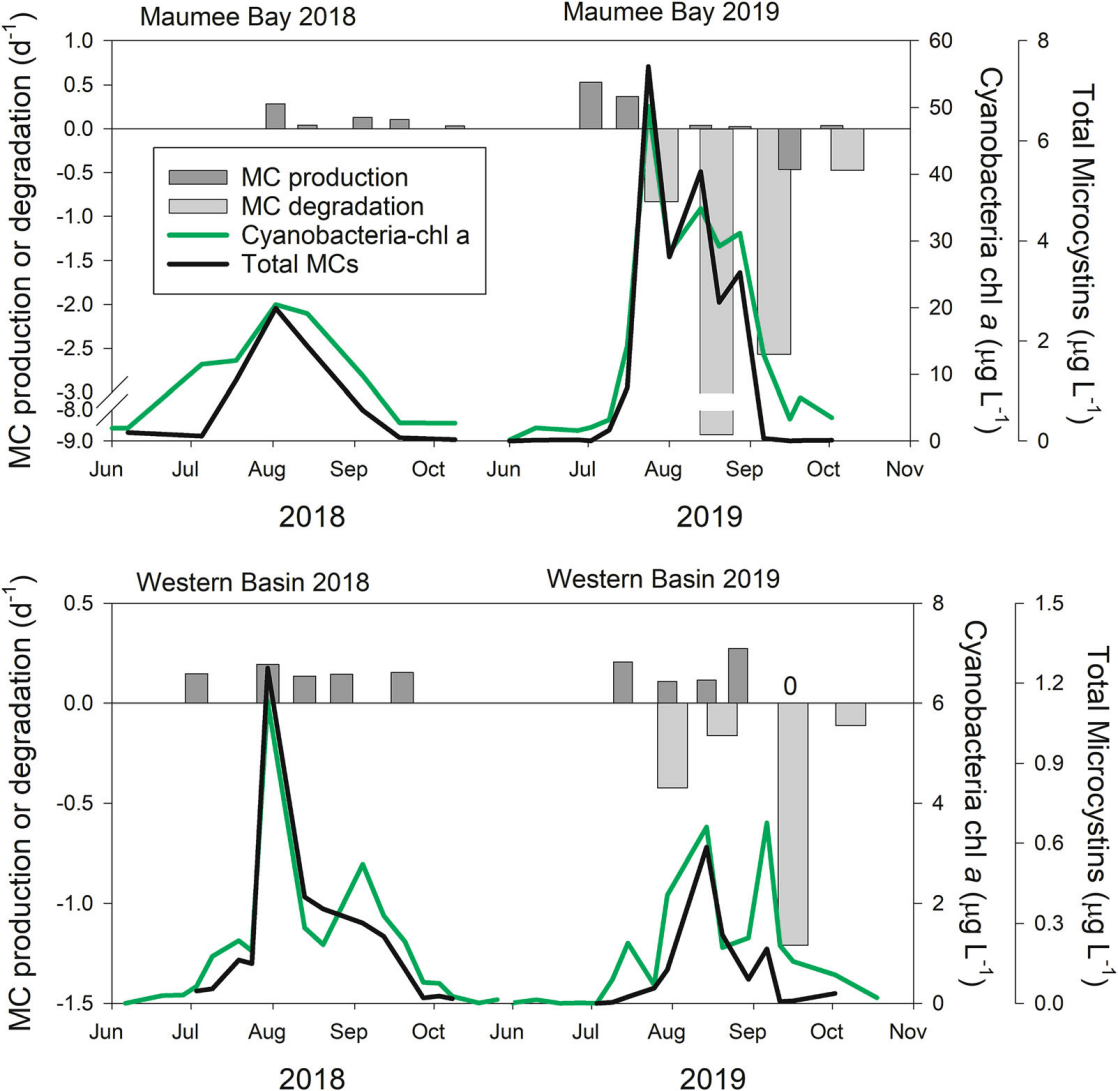
Seasonal pattern of microcystin (MC) production and biodegradation, cyanobacteria‐chlorophyll *a*, and concentration of total microcystins at two sites in western Lake Erie during 2018 and 2019. Microcystin production rates shown are an average of the control treatment mean (of three replicates) and the maximum mean of the phosphorus and nitrogen enrichment treatments. MC degradation data shown is the biotic treatment mean of three replicates. “0” on the MC production panel indicate zero MC production. Note the difference in *Y*‐axis scales.

The nearshore station MB18 exhibited greater levels of both cyanobacteria‐Chl *a* and microcystin concentrations than the offshore WB‐83 station (Fig. 2). Cyanobacteria‐Chl *a* concentration during 2018 at MB18 increased throughout July and peaked in early‐to‐mid August at 20.5 *μ*g L^−1^ and decreased throughout September (Fig. 2a), whereas cyanobacteria‐Chl *a* concentrations at WB‐83 were between 0.9 and 2.5 *μ*g L^−1^ throughout the summer except reaching 6.0 *μ*g L^−1^ on 30 July 2018 (Fig. 2b). Cyanobacteria‐Chl *a* concentrations were twice as high during 2019 at MB18 with concentrations exceeding 25 *μ*g L^−1^ from mid‐July to the end of August and peaked at 50.3 *μ*g L^−1^ in late July. Cyanobacteria‐Chl *a* concentration at WB‐83 ranged from 0.4 to 3.6 *μ*g L^−1^ during 2019.

Total microcystins concentrations followed a similar pattern as cyanobacteria‐Chl *a* concentrations, but the post‐peak decline was steeper (Fig. 2). Total microcystins at nearshore MB18 peaked at 2.65 and 7.48 *μ*g L^−1^ during 2018 and 2019, respectively (Fig. 2a). Total microcystins at WB‐83 peaked at 1.26 and 0.59 *μ*g L^−1^ during 2018 and 2019, respectively (Fig. 2b). Extracellular microcystins followed a similar pattern as total microcystins, but concentrations were less than 10% of total microcystins (Supporting Information Fig. S1).

The mass ratio of total microcystins to cyanobacteria‐Chl *a* at the nearshore site MB18 was highest in July and August of both years (0.1–0.2) and was lowest in September (< 0.02) (Supporting Information Fig. S1). At site WB‐83, microcystins to cyanobacteria‐Chl *a* showed a hump‐shaped pattern with the highest microcystins to cyanobacteria‐Chl *a* occurring in August (0.2–0.3), intermediate ratios in July and lowest in September (< 0.02).

Water temperature of incubation ranged from 21.5°C to 26.6°C for all experiments except those conducted in October of 2018, which were cooler (Table 1). Ambient nitrate concentrations at MB18, the nearshore site, (up to 181.9 *μ*mol L^−1^) were greater than WB‐83, the offshore site, and nitrate concentrations generally decreased throughout summer at both sites. Ammonium and urea‐N concentrations were much lower than nitrate, frequently below detectable levels, and no spatial or temporal pattern was observed. Dissolved reactive P (DRP) concentration at the nearshore site ranged from below detection to 1.45 *μ*mol L^−1^ with the highest concentrations occurring during early summer 2019. DRP concentrations were lower at the offshore site ranging from less than detectable levels to 0.19 *μ*mol L^−1^.

**Table 1:** The initial concentrations of cyanobacteria, particulate microcystins (MCs), nutrients, and incubation of 22 microcystin production experiments were conducted with water collected at two sites in western Lake Erie during 2018 and 2019.

Site	Date	Cyanobacteria‐Chl *a* (*μ*g L^−1^)	Part. MCs (*μ*g L^−1^)	Water temp. (°C)	Nitrate + NO_2_ (*μ*mol L^−1^)	Ammonium (*μ*mol L^−1^)	Urea‐N (*μ*mol L^−1^)	DRP (*μ*mol L^−1^)
MB18	02 Aug 18	20.5	0.869	21.80	73.40	<0.50	3.49	<0.03
MB18	14 Aug 18	19.2	3.292	24.19	26.06	<0.50	<1.00	0.06
MB18	04 Sept 18	10.9	0.520	23.84	39.97	1.14	<1.00	0.21
MB18	18 Sep 18	3.1	0.113	21.56	25.25	1.40	<1.00	0.19
MB18	09 Oct 18	2.7	0.028	17.68	28.43	6.01	<1.00	0.71
WB‐83	03 Jul 18	1.0	0.121	26.57	16.48	3.54	No data	0.12
WB‐83	30 Jul 18	7.2	2.621	24.93	21.95	1.39	No data	0.19
WB‐83	13 Aug 18	1.3	0.662	25.68	11.87	<0.50	<1.00	<0.03
WB‐83	27 Aug 18	0.5	0.322	24.60	10.71	0.75	<1.00	<0.03
WB‐83	19 Sep 18	1.7	0.086	23.67	5.10	1.02	<1.00	0.15
WB‐83	08 Oct 18	0.0	0.000	19.83	19.25	0.74	3.60	0.06
MB18	02 Jul 19	2.1	0.050	25.20	181.93	1.29	13.94	1.45
MB18	16 Jul 19	14.3	1.065	26.33	113.39	0.76	<1.00	0.43
MB18	13 Aug 19	34.9	5.384	24.58	0.66	1.05	<1.00	<0.03
MB18	28 Aug 19	31.2	3.370	22.01	38.86	3.14	<1.00	0.06
MB18	16 Sep 19	3.3	0.006	22.02	27.78	7.56	<1.00	0.52
MB18	02 Oct 19	3.5	0.016	20.98	23.89	5.05	2.47	0.31
WB‐83	13 Jul 19	1.6	0.042	25.52	50.35	0.74	4.92	<0.03
WB‐83	30 Jul 19	1.6	0.404	25.68	18.10	<0.50	<1.00	<0.03
WB‐83	14 Aug 19	7.5	1.425	24.86	5.99	1.24	<1.00	<0.03
WB‐83	26 Aug 19	1.3	0.195	23.40	9.24	<0.50	<1.00	<0.03
WB‐83	16 Sep 19	0.5	0.005	22.59	4.28	0.63	4.71	<0.03

Cyanobacteria‐Chl *a* and total microcystin concentration correlated significantly, primarily driven by the few high biomass and microcystins samples from the nearshore site (*p* < 0.001, *R* = 0.95, Supporting Information Fig. S2). The cyanobacteria‐Chl *a* and total microcystin concentrations relationships with nutrient concentration were pyramid‐shaped (Supporting Information Fig. S2): the highest cyanobacteria‐Chl *a* and total microcystin concentrations occurred at intermediate nutrient concentrations, but low cyanobacteria‐Chl *a* and total microcystin concentrations occurred early summer when nutrient concentrations were at their highest.

### Microcystin production experiments

**Fig. 3:**
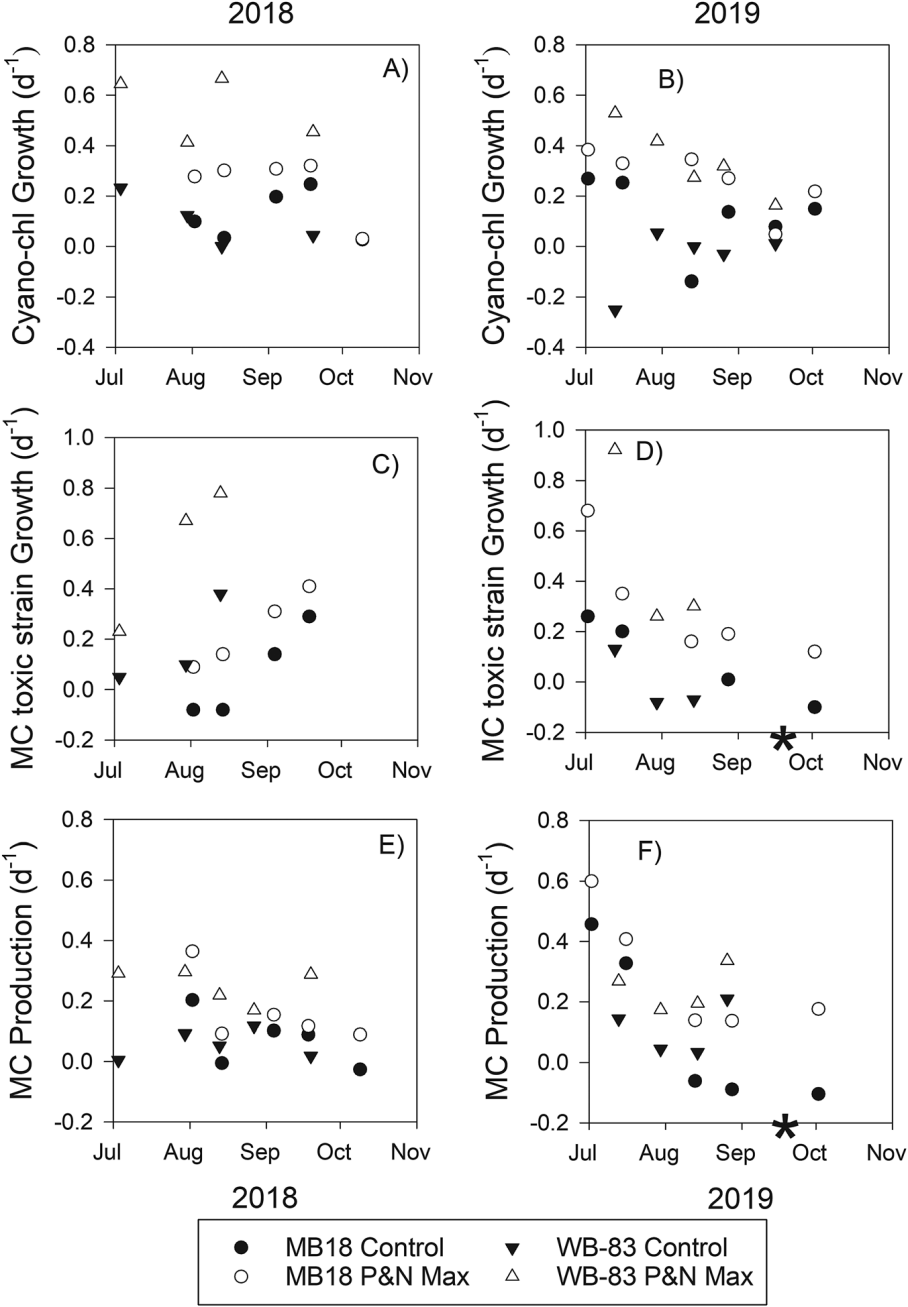
Microcystis‐dominated cyanobacterial‐chlorophyll *a* specific growth rate (**a** and **b**), microcystin toxic strain (based on *mcyE*) specific growth rate (**c** and **d**), and microcystin (MC) production rate constants (**e** and **f**) determined in experiments at nearshore site MB18 (circles) and site WB‐83 in the center of the western basin (triangles) in 2018 and 2019. Black symbols are mean of the ambient nutrient control and the white symbols are the maximum mean rate constants of the P‐ and N‐enriched treatments. The asterisk indicates the 16 September 2019 MB18 experiment when mcyE was not detected and microcystin production was negative in all treatments.

*Microcystis*‐dominated cyanobacterial specific growth rates (based on cyanobacteria‐Chl *a* concentration) in the controls (no nutrients added) ranged from −0.25 to 0.67 d^−1^ and averaged 0.08 ± 0.14 (± 1 standard deviation) d^−1^ across all experiments (Fig. 3). In every experiment, except the experiment conducted 16 September 2019 with the nearshore water, growth rates of the P and N enrichments were greater than the no nutrient enrichment control (three‐way ANOVA with Tukey test, *N* = 20, *p* < 0.001; Fig. 3a,b). Ammonium and P enrichments resulted in significantly greater growth (0.35 ± 0.16 d^−1^) than P and nitrate (0.22 ± 0.12 d^−1^) and urea (0.26 ± 0.13 d^−1^). Low solar radiation values during the first 2 days of deployment of the 16 September 2019 incubation likely contributed to zero growth. During 2018, high variability led to no apparent spatial or temporal patterns of growth rates over the season. During 2019, the growth rates of the P and N enrichments for both sites decreased throughout the summer and fall. The greatest growth rates among the controls were recorded during the July 2019 experiments with nearshore water.

Specific growth rates of toxic strains (based on the quantification of the *mcyE* gene) in the no nutrient controls were 0.06 ± 0.17 d^−1^ across all experiments, except in the 16 September 2019 nearshore experiment when *mcyE* was not detected. Toxic strain growth in the P and N enrichments were greater than the control in every experiment (*p* = 0.009; Fig. 3(c,d)). Tukey test indicated there was no significant difference among the N forms (*p* > 0.05), but P and ammonium (0.36 ± 0.25 d^−1^) and urea (0.35 ± 0.28 d^−1^) tended to result in greater toxic strain growth than nitrate (0.23 ± 0.23 d^−1^). There were no apparent seasonal patterns in toxic strain growth during 2018 due to high variability. During 2019, toxic strains growth rate in the controls and the P and N enrichments decreased from summer through fall. The early July 2019 experiments (the first two at the nearshore and the first at the offshore locations) had positive toxic strain growth rates in the controls ranging from 0.13 to 0.26 d^−1^, whereas the later experiments had toxic strains rate constants less than 0.01 d^−1^ in the controls.

Microcystin production in the no nutrient controls averaged 0.09 ± 0.13 d^−1^ across all experiments, and there was greater production in the P and N enriched treatments (*p* = 0.047; Fig. 3e,f). Like toxic strain growth, there was no statistical difference in microcystin production among the three forms of N (nitrate, ammonium, and urea) used in the P and N enrichment treatments (0.18 ± 0.10, 0.18 ± 0.13, and 0.21 ± 0.14 d^−1^, respectively). During 2018, microcystin production rate constants ranged from 0.01 to 0.36 d^−1^ with no apparent spatial or temporal pattern. During 2019, microcystin production rate constants of the controls and the P and N enrichments for both sites decreased throughout summer. Greatest microcystin production rate constants among the controls were recorded during the July 2019 experiments with nearshore water at 0.46 and 0.33 d^−1^.

Across all experiments there were significant positive correlations between toxic strains growth and microcystin production (*r* = 0.416, *p* < 0.01), cyanobacteria growth rate and toxic strains growth (*r* = 0.547, *p* < 0.01), and cyanobacteria growth and microcystin production (*r* = 0.307, *p* < 0.01) (Supporting Information Fig. S3). Furthermore, cyanobacterial growth and microcystin production in the P and N enrichments exceeded controls, but no difference was identified among the three N forms (Supporting Information Fig. S3 top row). Temporal trends suggested higher microcystin production per toxic strain growth and per cyanobacteria growth in July and lower microcystin production in August and September (Supporting Information Fig. S3 bottom).

### Microcystin biodegradation and incorporation experiments

Microcystins can be removed from the extracellular phase by two mechanisms: microbial biodegradation and adsorption onto clays. Measurements of ^15^N‐microcystin‐LR in the particulate phase (extracted from the filter) were always below the detection limit (method detection limit < 0.5 ng L^−1^). This suggests that (1) *Microcystis* cells did not reincorporate extracellular microcystin‐LR, and (2) extracellular microcystin‐LR did not stick to sediment particles and was thus likely degraded biologically.

Extracellular ^15^N‐microcystin‐LR concentrations in the abiotic controls remained stable throughout 48 h of incubation for all nine microcystin biodegradation experiments (Supporting Information Fig. S4). ^15^N‐microcystin‐LR concentrations in the biotic treatments decreased throughout incubation, but the loss rate differed among the experiments. The biotic treatments' degradation rate constants ranged from −0.11 to −8.76 d^−1^ (Fig. 2). In all experiments conducted, degradation at the nearshore site was faster than at the offshore site. There were no apparent correlations among biodegradation rate and water temperature, cyanobacterial biomass, or microcystin concentration (Supporting Information Fig. S5), but there could be a lag effect as the greatest biodegradation rate followed peak biomass and microcystin concentration (Fig. 2).

**Fig. 4:**
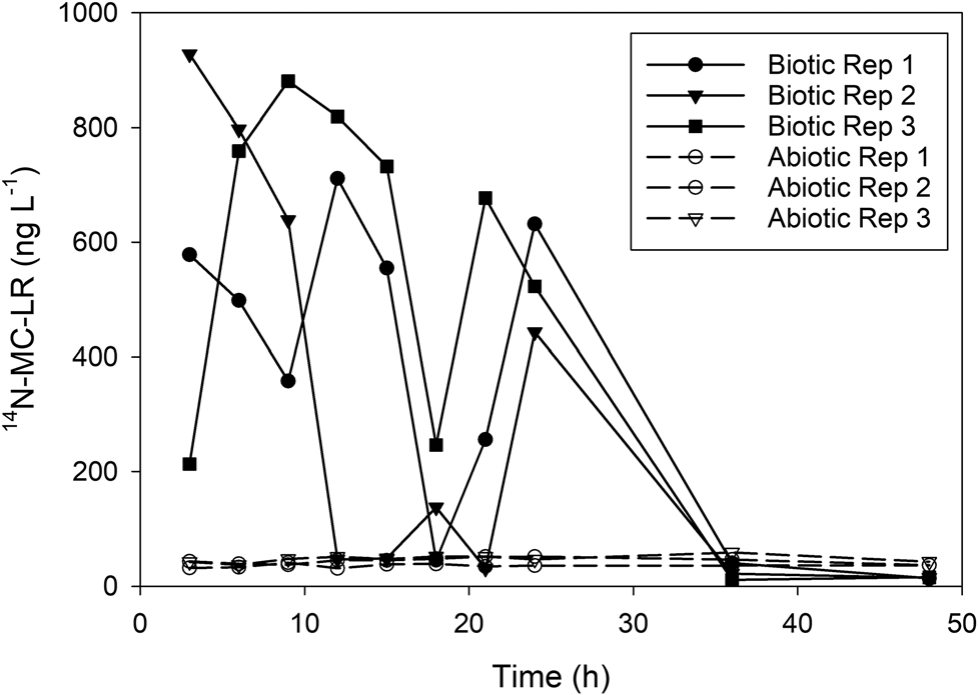
Extracellular concentrations of non‐isotope‐labeled (naturally occurring) microcystin‐LR during the 2019 degradation experiment conducted with nearshore site MB18 water on 19 August 2019. Solid lines are the biotic treatments and dashed lines are the abiotic treatments. Each line represents a biological replicate.

Naturally produced microcystin‐LR (detected as ^14^N microcystin‐LR by LC/MS and referred to as “unlabeled microcystin‐LR” below) was primarily cell‐associated. We did not detect extracellular unlabeled microcystin‐LR, except throughout the nearshore August 2019 experiment (Fig. 4). Extracellular unlabeled microcystin‐LR concentrations remained low and stable throughout the 48 h in the abiotic control. There were differences in unlabeled microcystin‐LR concentrations among the three biotic treatment replicates. One replicate had an unlabeled microcystin‐LR concentration of 213 ng L^−1^ at 3 h, and it increased to 880 ng L^−1^ by 9 h, indicating microcystin release from the cells. Another replicate had an unlabeled microcystin‐LR concentration of 928 ng L^−1^ at 3 h, and it declined to 46 ng L^−1^ by 12 h, indicating biodegradation. Between hours 18 and 21, unlabeled microcystin‐LR concentrations increased in all three biotic replicates and decreased again to low levels by hour 36. These findings demonstrate that extracellular microcystin‐LR production (from lysis events) and subsequent biodegradation can occur on very short timescales and can be quite variable even among replicates within the same experiment.

**Fig. 5:**
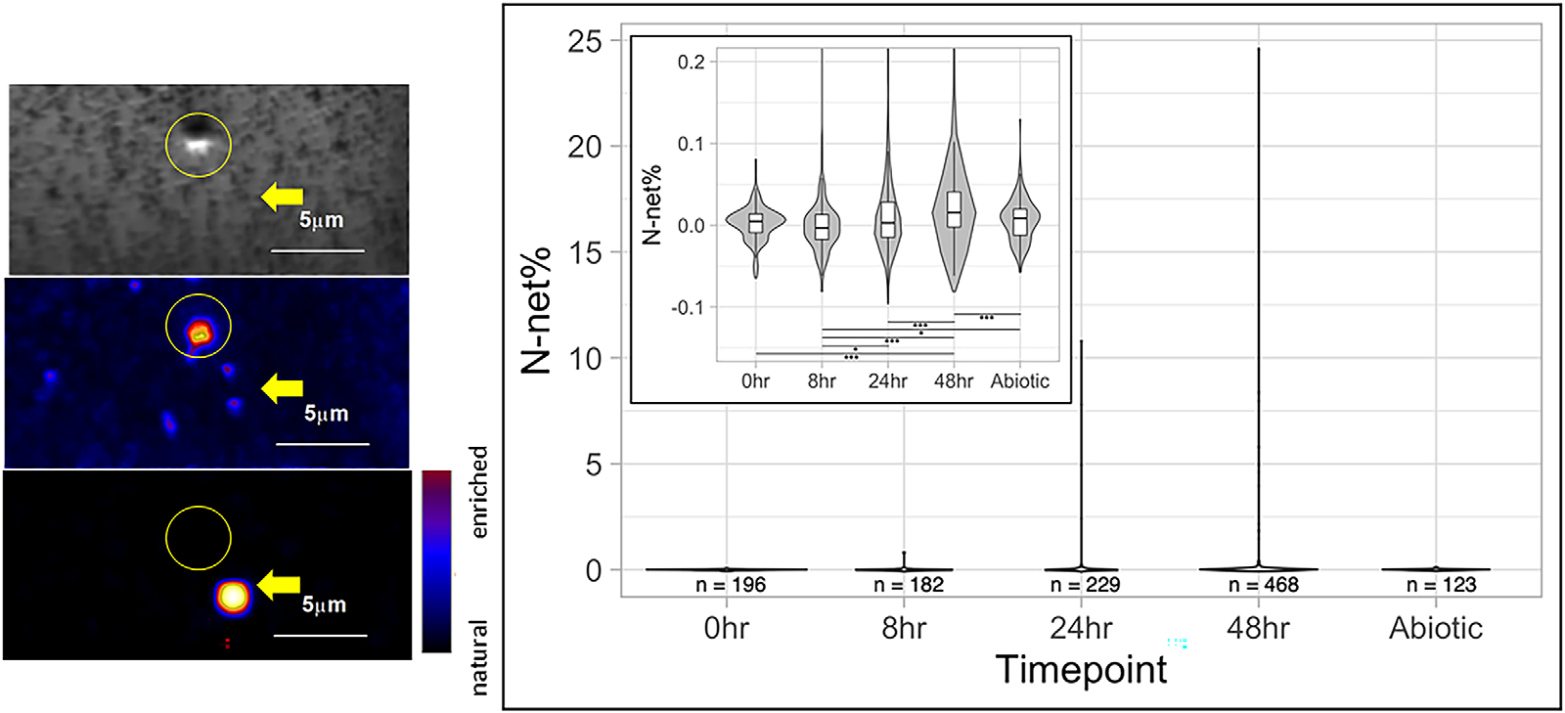
Single‐cell nitrogen incorporation using NanoSIMS analysis. Representative images on the left show secondary electron (topography, top), N‐containing organic biomass (middle), and ^15^N enrichment maps (bottom). A *Microcystis* sp. cell (circle) and a highly ^15^N labeled heterotrophic bacterial cell (arrow) are highlighted. Right panel: Nitrogen isotope data (N_net_, or percent of new biomass from microcystin ^15^N) for non‐*Microcystis* cells collected from incubations over time. Shown are ranges, medians, and 25^th^ and 75^th^ percentiles (inset shows different scale). Asterisks identify datasets that are statistically significantly different based on Wilcoxon rank‐sum test (* = 0.05 > *p* > 0.01; ***p < 0.001).

The 2020 single biodegradation and incorporation experiment showed similar results to the 2019 experiments, with negligible removal of ^15^N‐microcystin‐LR in the abiotic controls, 75% removal in the biotic treatment after 48 h, and undetectable ^15^N‐microcystin‐LR in the particulate fraction (Supporting Information Fig. S4). In the NanoSIMS analysis from the combined triplicate incubations, we collected data from ~ 200 heterotrophic cells at times 0, 8, and 24 h, and 468 heterotrophic cells at 48 h, where we expected the highest isotope incorporation (Fig. 5). The bacterial population from whole water samples collected 8, 24, and 48 h after ^15^N‐microcystin‐LR inoculation was statistically more isotopically enriched than the initial samples, and each was more enriched than the time point preceding it (Fig. 5). The isotope enrichment was mostly due to a small number of very highly enriched cells, rather than the entire population being enriched. This can be better conceptualized if we establish a cutoff of 1% N_net_, which would include cells that incorporated at least 1% of their N requirement from the added microcystin‐LR. After 24 h, only 1.7% (4/229) cells had a N_net_ greater than 1%, and after 48 h, 2.7% (13/468). The highest N_net_ recorded was a cell in the 48‐h sample with a N_net_ of 24%. We also analyzed 123 cells from the abiotic control 48‐h triplicate samples, which shows that some contaminant bacteria eventually grew in those incubations. However, the isotope signal of this population was indistinguishable from the initial samples, which was in agreement with the LC–MS/MS data showing negligible ^15^N‐microcystin‐LR removal. These contaminant bacteria clearly did not use the added ^15^N‐microcystin‐LR as a source of nitrogen.

We also examined the isotope incorporation of the majority of the cells in the particulate fraction from the biotic incubations (Fig. 5, inset). At the population level, these cells also increased in ^15^N incorporation over time, showing that some of the N from microcystin‐LR eventually made its way into many of the microbial cells in the lake. However, the N_net_ median value, even after 48 h, was very low (0.01%), showing that the N incorporation, while statistically detectable, was biogeochemically negligible. A similar pattern emerged for the ^15^N incorporation by *Microcystis*‐like cells (Supporting Information Fig. S6). Here again, the populations were statistically different, for example, *Microcystis*‐like cells after 48 h were more ^15^N enriched than cells from the 8‐h timepoint, but N_net_ values were also extremely low (maximum 0.04%, median 0.005% after 48 h). These data suggest that *Microcystis* cells likely did not incorporate (or adhere to) extracellular microcystin‐LR, and N remineralized by microcystin‐LR‐degrading bacteria did not support much *Microcystis* N demand.

## Discussion

### Microcystin production

We tracked microcystin production at two sites throughout two bloom seasons in western Lake Erie, which had very different biomass and microcystin concentrations (Fig. 2). The hypothesis that there would be greater rate constants of microcystin production in the early bloom stages was supported for the larger 2019 bloom but not supported by the experiments conducted during the 2018 smaller bloom (Fig. 2). However, no experiments were conducted during July 2018 when the nearshore site may have had higher production rates as toxin concentrations generally are elevated at the beginning of the bloom and decrease as the bloom season progresses (Gobler et al. 2016).

Microcystin production in the P and N dual‐amended treatments exceeded the controls in every experiment, indicating that these nutrients limited microcystin production throughout the 3‐d incubations, which has been shown before for Lake Erie (Chaffin et al. 2018; Jankowiak et al. 2019) and in *Microcystis* blooms elsewhere (Davis et al. 2010). The ambient nitrate and P concentrations at nearshore MB18 during July 2019 exceeded the enrichment concentrations, and slight differences in microcystin production between the control and the P and N addition treatments were observed, indicating nutrients were less limiting in those early summer experiments. Larger differences between the control and the P and N addition treatments were recorded when ambient concentrations were lower later in the season. However, the maximum microcystin production in the P and N treatments measured during late summer and fall did not reach the same production as the early summer, indicating factors besides nutrients constrain microcystin production during late summer and fall.

In addition to the nutrient effect on microcystin production, there was also a seasonality effect. There are several possible factors that could explain lower microcystin production in the late season. A seasonal transition in the *Microcystis* community from mostly potentially toxigenic strains to mostly nontoxic stains in Lake Erie has been correlated to lower N availability (Gobler et al. 2016; Kitchens et al. 2018), which results in lower microcystins to biomass ratios during the late season (Supporting Information Fig. S1; Gobler et al. 2016). Correspondingly, toxic strain growth rates were lower during the late season (Fig. 3). Furthermore, the early growth phases of *Microcystis* in culture have greater *mcyE* transcription rates than the stationary phase (Rueckert and Cary 2009), which aligns with higher microcystin production detected in the early season in our study. Also, greater light intensity and longer photoperiods in early summer can lead to higher microcystin production (Kaebernick et al. 2000) and more toxic strains (Kardinaal et al. 2007). It is likely that the *Microcystis* community composition, light and nutrient availability, and growth phases all interacted to drive higher microcystin production rates during the early bloom and then lower production rates during the later bloom stages.

Finally, we did not attempt to normalize microcystin production to chlorophyll or another metric of cyanobacterial biomass. Cyanobacteria‐specific Chl *a* concentrations increased faster than did microcystins, which would give the inaccurate impression that microcystin production was decreasing or negative if normalized to biomass. The fact that *Microcystis* growth outpaced microcystin production follows the growth‐differentiation balance hypothesis that states autotrophs will shunt resources away from secondary metabolism toward growth and cellular division (Herms and Mattson 1992; Heath et al. 2016). The relationship between microcystin production and growth rate can be seen in the seasonal pattern of the microcystin‐to‐Chl *a* ratio (Supporting Information Fig. S1). In addition, we could not determine if the increase in microcystin was due to upregulation of *mcy* genes (Harke and Gobler 2015; Chaffin et al. 2018), more toxic strains producing a steady amount of microcystin (Davis et al. 2010), or both. Microcystin production rate constant is independent of cyanobacteria biomass and community composition, and therefore, can be incorporated into forecasts if initial microcystin concentrations are known.

### Microcystin biodegradation and incorporation

We are not aware of previous studies linking microcystin production and biodegradation in a naturally occurring *Microcystis*‐dominated cyanobacterial bloom. The results of our study provide evidence for in situ microbial degradation of microcystin. To our knowledge, using ^15^N isotope labeling to trace the fate of an algal toxin into the bacterial community has not been accomplished prior to our study, and a major finding was that degradation appears to be limited to a small subset of the Lake Erie microbial community. Our LC–MS/MS experiments clearly showed that ^15^N‐microcystin‐LR was removed in the whole water biotic incubations and remained stable in the abiotic controls throughout the 2019 experiments and the 2020 experiment, and the NanoSIMS analyses of the 2020 experiment showed incorporation by non‐*Microcystis* (presumably heterotrophic) cells. Data from these two independent experiments using different methods suggest that removal was due to microbial degradation rather than adhesion to mineral particles. First, if microcystin removal was caused by binding to clays, we would likely have detected ^15^N‐microcystin‐LR in the particulate fraction of the incubations, which we did not. Both the abiotic controls and the biotic samples retained < 1% of the ^15^N‐microcystin‐LR in the particulate fraction. Since the retained ^15^N‐microcystin‐LR was similar in both the abiotic control and the biotic treatment, these data suggest the filter adsorbed minimal quantities of ^15^N‐microcystin‐LR. Second, our NanoSIMS analyses identified ^15^N isotope incorporation associated with a small portion of the bacteria‐sized cells suggesting that (1) ^15^N‐microcystin‐LR was not adsorbed to mineral particles (we would have detected those as low C and N containing areas with ^15^N isotope enrichment), (2) ^15^N‐microcystin‐LR degradation was not carried out by the *Microcystis* cells but rather by smaller, presumably heterotrophic, cells, and (3) that the first step in microcystin degradation occurs very quickly once it gets into the cells. Furthermore, the NanoSIMS data support the hypothesis that a small number of heterotrophic bacterial taxa, likely representing multiple species, perform microcystin degradation (Fig. 5). We hypothesize that the heterotrophic bacteria that degrade microcystin‐LR either increased in abundance or their cell‐specific degradation activities because the highest microcystin biodegration rates followed peak bloom conditions (Fig. 2).

The literature on bacteria‐mediated microcystin degradation dates back to the 1990s (Jones et al. 1994; Dziga et al. 2017), and two primary pathways, the *mlr* ^+^ *and mlr* ^−^, have been identified for microcystin biodegradation (Bourne et al. 2001; Santos et al. 2021). The *mlr* ^+^ biodegradation has been linked genetically to *Sphingophyxis* sp. and others, whereas the *mlr* ^−^ biodegradation has been linked to *Paucibacter toxinivorans* without the identification of a gene cluster. Recent microcystin biodegradation investigations have focused on Lake Erie waters, suggesting a different *mlr* ^−^ degradation pathway exists, since there are bacteria that can degrade microcystin that do not have the *mlr* ^+^ pathway (Thees et al. 2019) and there is no evidence of *P. toxinivorans* in Lake Erie (McCartney et al. 2020). Since the microcystin degradation pathway(s) utilized by Lake Erie microbes are unknown, the questions of how the bacteria degrade microcystin‐LR and which components of the molecule are incorporated in the bacterial biomass remain unanswered.

Microcystin degradation (Fig. 2) was faster at the nearshore site than at the offshore site. At both sites, the highest microcystin degradation rates followed peak biomass and microcystin concentrations, which suggests that microcystin‐degraders likely respond to *Microcystis* biomass, microcystin production, microcystin release, or a combination of these factors. Previous research from a eutrophic lake in Spain found a positive correlation between microcystin‐biodegrading bacteria and microcystin concentrations (Lezcano et al. 2016). The microcystin degrading community composition can be influenced by dissolved inorganic carbon and nitrogen concentrations (Giaramida et al. 2013), and these environmental drivers can have opposite impacts on *mlr* ^+^ and *mlr* ^−^ communities (Li et al. 2011). In a 2017 study, the microcystin degradation rate constant reported for the *P. toxinivorans*, *mlr* ^−^ system was − 1.3 d^−1^ whereas the microcystin‐LR degradation rate constant for the *mlr* ^+^ system was − 10 d^−1^ (Morón‐López et al. 2017), which is similar to our observed value. Kansole and Lin (2016) have reported a different *mlr* ^−^ pathway by *Bacillus* sp., with low rate constant (− 0.4 d^−1^). In our incubations, Lake Erie microcystin‐LR degradation rate constants were the most negative (more negative values indicate higher degradation) following the bloom peak and were much greater than those reported from pure cultures containing the *mlr* ^−^ pathways, suggesting a complete microbial ecosystem is needed for efficient microcystin‐LR degradation. Furthermore, NanoSIMS data suggest that microcystin‐LR degrading cells used N from the extracellular microcystin‐LR at environmentally realistic concentrations for their own N demand rather than making that N available to the rest of the microbial community.

Dissolved naturally occurring microcystin‐LR (> 99% being ^14^N‐microcystin‐LR) was monitored in parallel with the added ^15^N‐microcystin‐LR. Specifically, the experiment with the highest biodegradation rate (nearshore, 19 August 2019) had quantifiable concentrations of dissolved natural microcystin‐LR (Fig. 4) that were released by either cell lysis or cross membrane transport from *Microcystis*. In this experiment, the added ^15^N‐microcystin‐LR was depleted to below detection within 15 h, and the natural microcystin‐LR degradation rate constant was − 10 d^−1^ over the first 24 h, similar to the ^15^N‐microcystin‐LR degradation rate constant of − 8.8 d^−1^. Release of natural microcystin‐LR occurred between 21 and 24 h in all three microcosms and was then subsequently degraded with a smaller rate constant of − 3.6 d^−1^. The decrease in the microcystin‐LR degradation rate constant over the last 27 h may have been caused either by a decrease in microbial activity and/or a release of microcystin‐LR over this time. If the decrease was solely due to a release of microcystin‐LR from the cells, the cellular lysis rate constant would be estimated at − 7 d^−1^. Lysis (release) events in natural environments are generally associated with cyanophage attacks (Steffen et al. 2017; McKindles et al. 2020). These potential mechanisms need to be further investigated, but here we demonstrate the power of using ^15^N‐microcystin‐LR additions to track metabolism in a complex microbial community in a natural bloom.

### Improving predictions of microcystin concentrations

Current forecasts of cyanobacterial HABs in Lake Erie rely on remote sensing biomass data and water current models to transport the biomass (Rowe et al. 2016), and they do not incorporate microcystins. Forecasts of microcystins concentrations would be critical to drinking water treatment plants and beach managers when making decisions on water treatment and beach closures, respectively. For example, the Ohio EPA has thresholds of 0.3 and 1.6 *μ*g L^−1^ for children and adult safe drinking water (based on the ELISA method; Ohio EPA 2020), and U.S. EPA threshold for recreation contact is 8.0 *μ*g L^−1^ (U.S. EPA 2019). Recent reports have attempted to hindcast microcystin concentrations using remote sensing biomass data and microcystin‐to‐Chl *a* ratio from weekly grab samples to develop forecasts that give the probability of exceeding a particular health guideline, but these models did not account for cyanobacteria growth, microcystin production, or microcystin loss (Liu et al. 2020; Qian et al. 2021). It would be advantageous not to treat microcystins as inert particles that are only transported but incorporate net microcystin production to allow the microcystin to increase or decrease given the environmental conditions.

The variable microcystin production that we detected raises the question of what microcystin production rate constants should be incorporated into models. The “actual” rate constants that occurred in the lake would have likely been in between the control and the P and N amended treatments. One option is to use the average of the control and the P and N maximum rate (Fig. 2), and to use rates for MB18 for the area within 20 km of the mouth of the Maumee River (Fang et al. 2019) and WB‐83 rates for the rest of the western basin. Furthermore, we only quantified production rate constants every 2 weeks during the growing season, which leaves temporal gaps. While more research into the environmental factors regulating microcystin production and biodegradation is needed, our work demonstrates that greater microcystin production occurs in the early bloom season and greatest microcystin biodegradation occur at peak bloom. This information can be used to inform predictive models.

## Conflict of Interest

None declared.

## Supporting information


**Appendix S1** Supplementary Information

## Data Availability

Lake Erie field data can be found on Stone Lab and Ohio Sea Grant's research website at: https://ohioseagrant.osu.edu/research/live/water. All other data are available from the authors upon request.
