# Supplementary material for: Quantification of microcystin production and biodegradation rates in the western basin of Lake Erie
Source: Limnol Oceanogr. 2022 May 4;67(7):1470–83. doi: 10.1002/lno.12096 (PMC9543754; doi:10.1002/lno.12096)
Supplement: Supplementary file 1 — Appendix S1 Supplementary Information [file LNO-67-1470-s001.docx]

Supplementary Information for

Quantification of microcystin production and biodegradation rates in the western basin of Lake Erie

Justin D. Chaffin^a^*, Judy A. Westrick^b^, Elliot Furr^b^, Johnna A. Birbeck^b^, Laura A. Reitz^c,1^, Keara Stanislawczyk^a^, Wei Li^d^, Peter K. Weber^d^, Thomas B. Bridgeman^e^, Timothy W. Davis^c^, Xavier Mayali^d^

a: F.T. Stone Laboratory and Ohio Sea Grant, The Ohio State University, 878 Bayview Ave. P.O. Box 119, Put-In-Bay, OH 43456, USA

b: Lumigen Instrument Center, Wayne State University, 5101Cass Ave., Detroit, MI 48202, USA

c: Department of Biological Sciences, Bowling Green State University, Life Sciences Building, Bowling Green, OH 43402, USA

d: Nuclear and Chemical Sciences Division, Lawrence Livermore National Laboratory, Livermore CA 94550, USA

e: Lake Erie Center, University of Toledo, Oregon, OH, 43416, USA

1: Current address - Department of Earth and Environmental Sciences, University of Michigan, 2534 North University Building, 1100 North University Avenue, Ann Arbor, MI 48109-1005, USA

***Materials and methods***

**Water collection for experiments**

Lake water containing the natural assemblage of plankton was collected with a consistent methodology for both the microcystin production and biodegradation experiments; however, collection methods differed by site. Experimental water at site MB18 was collected with a two-meter-long PVC food-grade hose with a submersible pump that was lowered and raised at a steady rate (hand-over-hand) throughout the water column. Lake water was pumped into two 20-L lake water-rinsed carboys. At site WB-83, surface water was collected with a clean lake water-rinsed bucket and deposited into lake water-rinsed carboys, splitting each pull between carboys. Cyanobacterial surface scums can be very dense at MB18 (chlorophyll *a* > 500 µg L^-1^ at the surface), whereas scums are usually much less dense at WB-83. High biomass can result in extreme bottle effects (Barnard et al. 2021); therefore, the integrated water sample was taken from MB18 to dilute potential surface scums with underlying water which helped prevent experiments from starting with excessively high biomass.

**Cyanobacteria biomass**

Phytoplankton community composition and cyanobacterial biomass were quantified immediately using a FluoroProbe (BBE moldaenke, Germany) equipped with a bench-top cuvette reader, as previously described (Chaffin et al. 2013). The FluoroProbe uses chl *a* and accessory pigment fluorescence to quantify the chl *a* concentrations associated with four functional algal groups (green algae, cyanobacteria, diatoms, and cryptophytes) (Beutler et al. 2002). Previous research has shown total chl *a* and cyanobacteria-specific-chl *a* concentrations of bioassay experiments measured with the FluoroProbe agreed well with traditional filtered-extracted chl *a* and phycocyanin concentrations (*r*^2^ = 0.85, 0.93, respectively (Chaffin et al. 2013); therefore, we did not measure chl *a* collected on a filter. The western basin’s cyanobacterial community is historically dominated by *Microcystis* (Steffen et al. 2014), which also was the dominant cyanobacterial genus in our experiments (qualitative microscopy assessments of initial conditions for all experiments). The FluoroProbe cyanobacteria-chl *a* concentration data was used to calculate *Microcystis*-dominated cyanobacteria specific growth rates.

**Nutrient concentrations**

A 35 mL subsample from the initial carboy and each experimental bottle post-incubation was filtered (0.45µm) for dissolved nutrients (nitrate, ammonium, urea, and dissolved reactive P) and stored in a 60 mL PETG bottle frozen at -20°C until analysis. Nitrate, ammonium, and dissolved reactive P were analyzed with colorimetric methods on a SEAL Analytical Quaatro segmented flow auto-analyzer following standard methods (Chaffin et al. 2019). Urea (as urea-N) was quantified spectrophotometrically following the diacetyl monoxime with thiosemicarbazide (Chaffin and Bridgeman 2014).

**Microcystin analysis**

Liquid chromatography with tandem mass spectrometry (LC-MS/MS) was used to quantify total microcystins. 12 MC congeners ([D-Asp^3^]-MC-RR, MC-RR, MC-YR, MC-HtyR, MC-LR, [D-Asp^3^]-MC-LR, MC-HilR, MC-WR, MC-LA, MC-LY, MC-LW, and MC-LF) (Birbeck et al. 2019b). The detection limit varied between congeners with a range of < 0.5 to 5.0 part per trillion. Total MC concentration was determined as the sum of all 12 MC congeners, and microcystins concentrations in the bottles were back-calculated to account for both the volume of sample water filtered and the volume of deionized water.

**qPCR**

DNA was collected by filtering 20-50mL of sample water onto a 1.2µm polycarbonate filter and frozen at -80°C until extraction and analysis. DNA extraction was conducted with the QIAgen dNeasy™ and Blood & Tissue™ Kits (Qiagen, Carlsbad, CA, USA) using manufacturer’s methods (QIAgen dNeasy Standard Operating Procedure #001). After extraction DNA concentration and quality was measured using a NanoDrop Lite Spectrophotometer (Thermo Fisher Scientific Inc., Waltham, MA, USA). Analysis of DNA samples by qPCR for microcystin-producing cyanobacteria followed the protocol of Phytoxigene™ CyanoDTec multiplex assay (Diagnostic Technology, Birmingham, AL, USA), which is used by Ohio EPA for routine monitoring of cyanotoxins (Ohio EPA, 2018b).

Analysis of DNA samples for microcystin-producing cyanobacteria followed the protocol of Phytoxigene™ CyanoDTec multiplex assay (Diagnostic Technology, Birmingham, AL, USA), which is used by Ohio EPA for routine monitoring of cyanotoxins (Ohio EPA, 2018b). This protocol quantified the *mcyE* gene, one of ten genes in the *mcy* operon. Amplification was conducted on a Q- 4 channel qPCR system (Quantabio, Beverly, MA, USA) in a total volume of 25 μL. Individual reactions contain 5 µL of DNA sample and 20 μL of either Total (16S) CyanoDTec or Toxin CyanoDTec Master Mix (Phytoxigene™). Negative controls, consisting of PCR-grade water in place of DNA template, were run with each batch. A standard curve was created for the targeted *ndaF/mcyE* genes (Al-Tebrineh et al. 2012) using CyanoNAS nucleic acid standards (Phytoxigene™). Gene copies were calculated using cycle threshold (Ct) values with Quantabio software and back-calculated to gene copies L^-1^.

**^15^N-microcystin-LR**

A culture of *Microcystis* sp. was grown in Z8 medium with ^15^N-nitrate to create ^15^N-labeled microcystin-LR. ^15^N-microcystin-LR began to be harvested after 10 transfers of *Microcystis* into fresh ^15^N-nitrate Z8 medium. ^15^N-microcystin-LR was harvested by centrifugation and was isolated using solid phase extraction followed by semi-preparative high performance liquid chromatography with a photodiode array detector (Birbeck et al. 2019a). High resolution mass spectrometry was used to confirm that over 97% of the 10 N atoms in microcystin-LR were ^15^N. The ^15^N-microcystin-LR was used as a spike to differentiate between this added microcystin and the ambient microcystins, because the ambient microcystins could change throughout the experiment due to production, cellular lysis, and biodegradation. Differentiation between the isotope-labeled and natural abundance microcystins allowed for a more accurate calculation of biodegradation rates. Additionally, the fate of the ^15^N-microcystin-LR degradation products can be subsequently tracked into the microbial size fraction. To our knowledge, this was the first study to quantify microcystin biodegradation rates using ^15^N labeling.

**NanoSIMS**

To quantify bacterial incorporation of ^15^N_10_-microcystins-LR from the 2020 incubation, we collected cells from the degradation experiment at time points, 0, 2, 8, 24, and 48 hours. Isotope labeled incubation samples were fixed with 2% formaldehyde, stored overnight at 4°C, and filtered onto 0.2 µm white polycarbonate filters. Isotope imaging was performed with a CAMECA NanoSIMS 50 at Lawrence Livermore National Laboratory. The primary ^133^Cs+ ion beam was set to 2 pA, corresponding to an approximately 150 nm beam diameter at 16 keV. Rastering was performed over 20 x 20 μm analysis areas with a dwell time of 1 ms pixel^-1^for 19-30 scans (cycles) and generated images containing 256 x 256 pixels. Sputtering equilibrium at each analyses area was achieved with an initial beam current of 90 pA to a depth of ~60 nm, thus ensuring analysis of intracellular isotopic material. After tuning the secondary ion mass spectrometer for mass resolving power of ~7000, secondary electron images and quantitative secondary ion images were simultaneously collected for ^12^C^14^N^-^ and ^12^C^15^N^-^ on individual electron multipliers in pulse counting mode. All NanoSIMS datasets were initially processed using L’Image ([http://limagesoftware.net](http://limagesoftware.net/)) to perform deadtime and image shift correction of ion image data before creating ^12^C^15^N/^12^C^14^N ratio images, which reflected the level of ^15^N_10_ incorporation into biomass. Regions of interest (ROIs) for isotopic ratio quantification were drawn manually around each cell or using the automated particle ROI creation using L’Image and exported for statistical analyses in R version 4.02 (R core team, 2017). The fraction of cellular N incorporated from the substrate was calculated based on the time of incubation and initial enrichment of the labeled substrates (Popa et al. 2007). Xnet % is a measure of the newly synthesized biomass relative to the final biomass for a given element X, e.g., Nnet % = [*F*s/(*F*s + *F*i)] × 100, where *F*s is the fraction of N derived from the isotopically spiked substrate, and *F*i is the fraction of N from the original biomass. *F*s and *F*i are defined mathematically in (Popa et al. 2007), and are dependent on the final and initial isotopic ratios of the cell, as well as the isotopic ratio of the isotopically spiked substrate.

Cells from the biotic treatment (as well at hour 48 samples from the abiotic treatment) were filtered onto 0.2 µm membranes and their isotope signal was quantified by NanoSIMS to calculate the fraction of each cell’s biomass N that originated from the added ^15^N-microcystin-LR (Dekas et al. 2019). Note that the NanoSIMS secondary electron images enabled us to differentiate *Microcystis*-like cells from other cells (Fig. 5 in the main document), thus we report our data as non-*Microcystis* cells and *Microcystis*-like cells, with the assumption that the majority of non-*Microcystis* cells were heterotrophic, non-cyanobacterial cells, and we refer to them as bacteria herein, though they could also be archaea and picoeukaryotes.

***Results***

*Supplemental Figure 1. Cyanobacteria-chl a, total microcystins (MCs), the ratio of total microcystins to cyanobacteria-chl a, and extracellular microcystins at the nearshore site (MB18) and in the center of the western basin (WB-83) during 2018 and 2019.*


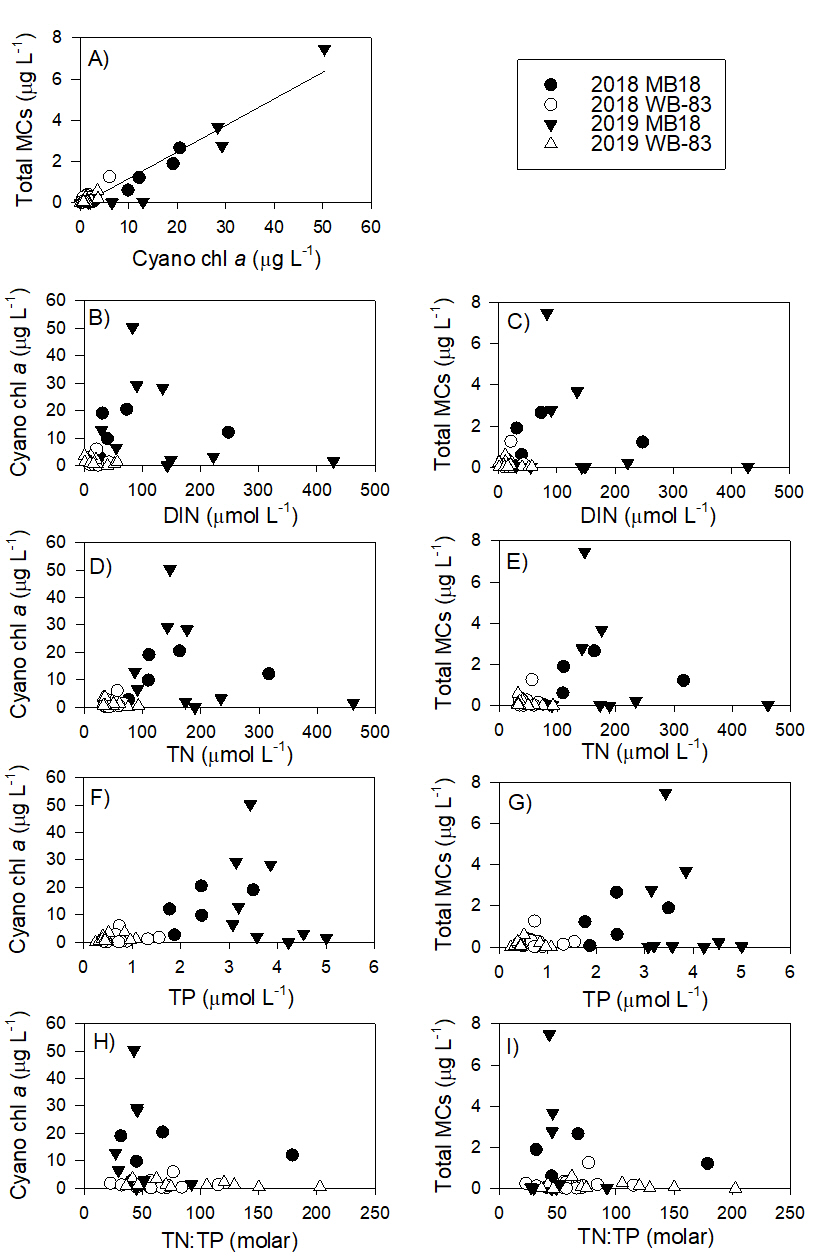


*Supplemental Figure 2. The correlation between cyanobacteria-specific chl a (cyano chl a) and total microcystins (MCs) was significant (panel A; P<0.001; R = 0.96), and the relationships between cyanobacteria-specific chl a and total microcystins (MCs) with the concentrations of dissolved inorganic nitrogen (DIN, the sum of nitrate, nitrite, ammonium, B & C), total nitrogen (D & E), total phosphorus (F & G), and the ratio of total nitrogen to total phosphorus (H & I) at the nearshore site (MB18) and in the center of the western basin (WB-83) during 2018 and 2019.*


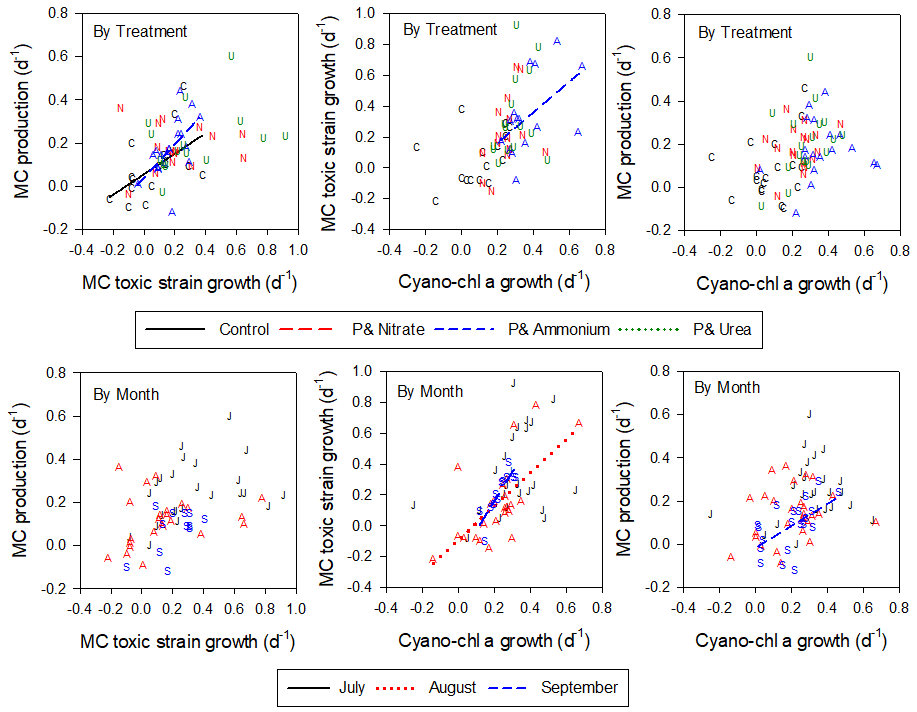


*Supplemental Figure 3: Scatter plots displaying the correlations among cyanobacteria-chl a growth, MC toxic strain growth, and microcystin production rate* *constants by treatment (top row; C=control, N = P&Nitrate, A = P&Ammonium, U = P&Urea) and month (bottom row; J = July, A = August, S = September and October). Icons show the treatment mean for each experiment. While the entire dataset correlations were significant for MC production vs. MC toxic strain growth (P = 0.009, R = 0.41), cyanobacteria-chl a growth vs. toxic strain growth (P < 0.001, R = 0.54), and cyanobacteria-chl a growth vs. MC production rate (P = 0.001, R = 0.36), not all correlations by treatment and by month were significant. Only the significant correlations (P < 0.05) have a trend line displayed in the by treatment and by month panels.*

*Supplemental Figure 4. Extracellular concentrations of ^15^N-microcystin-LR in the nine microcystin biodegradation experiments by season. Open icons with dashed lines are the abiotic control and the filled icons with solid line are biotic treatments. Icons are the treatment mean ± 1 standard error (of three replicates). In all experiments the ^15^N-microcystin-LR concentrations in the abiotic control were steady indicating no abiotic degradation while the concentrations in the biotic treatments decreased indicating biodegradation.*


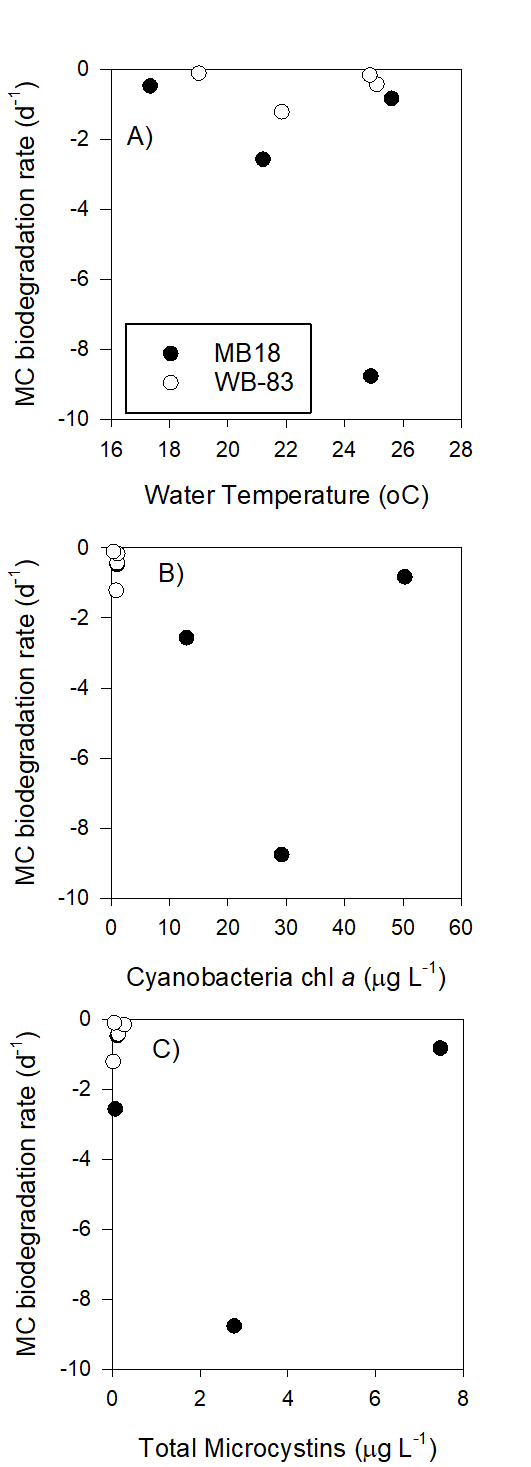


*Supplemental Figure 5. The relationship among the mean microcystin (MC) biodegradation rate and water temperature (A), cyanobacterial biomass as cyanobacteria-specific chlorophyll a (B) and ambient total microcystins (C) by site.*

*
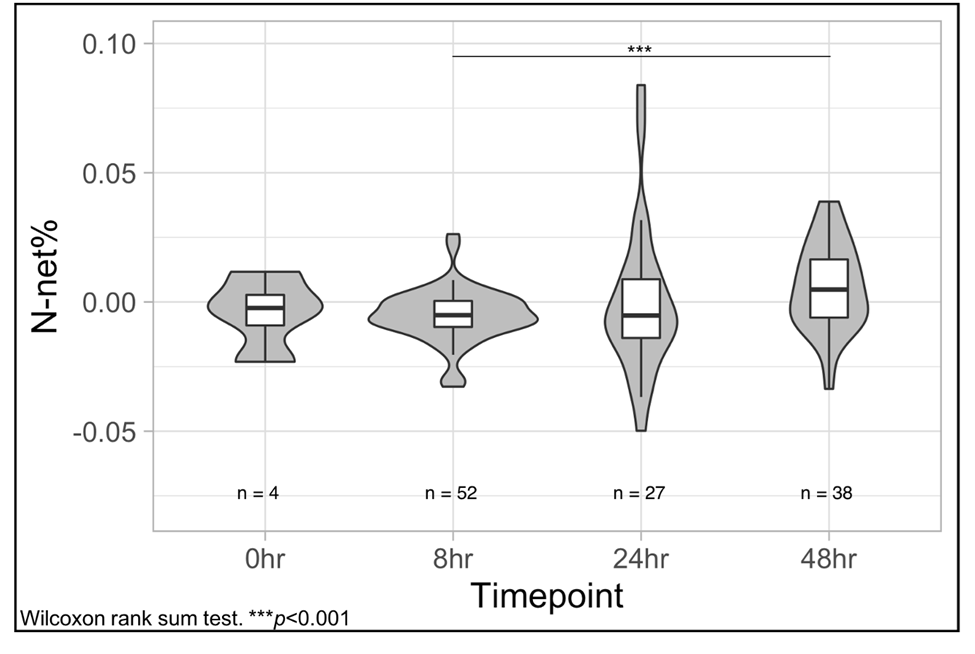
*

*Supplemental Figure 6: Nitrogen isotope data (N_net_, or percent of new biomass from microcystin N) derived from NanoSIMS analysis for Microcystis cells collected from incubations. Shown are ranges, medians, and 25th and 75th percentiles. Asterisks identify treatments at 8 hour and 48 hour were significantly different from one another based on Wilcoxon rank sum test (p < 0.001).*

Literature Cited

Al-Tebrineh, J., L. A. Pearson, S. A. Yasar, and B. A. Neilan. 2012. A multiplex qPCR targeting hepato- and neurotoxigenic cyanobacteria of global significance. Harmful Algae **15**: 19–25. doi:10.1016/j.hal.2011.11.001

Barnard, M. A., J. D. Chaffin, H. E. Plaas, and others. 2021. Roles of Nutrient Limitation on Western Lake Erie CyanoHAB Toxin Production. Toxins **13**: 47. doi:10.3390/toxins13010047

Beutler, M., K. H. Wiltshire, B. Meyer, C. Moldaenke, C. Lüring, M. Meyerhöfer, U. P. Hansen, and H. Dau. 2002. A fluorometric method for the differentiation of algal populations in vivo and in situ. Photosynth. Res. **72**: 39–53.

Birbeck, J. A., N. J. Peraino, G. M. O’Neill, J. Coady, and J. A. Westrick. 2019a. Dhb Microcystins Discovered in USA Using an Online Concentration LC–MS/MS Platform. Toxins **11**: 653. doi:10.3390/toxins11110653

Birbeck, J. A., J. A. Westrick, G. M. O’Neill, B. Spies, and D. C. Szlag. 2019b. Comparative Analysis of Microcystin Prevalence in Michigan Lakes by Online Concentration LC/MS/MS and ELISA. Toxins **11**: 13. doi:10.3390/toxins11010013

Chaffin, J. D., and T. B. Bridgeman. 2014. Organic and inorganic nitrogen utilization by nitrogen-stressed cyanobacteria during bloom conditions. J. Appl. Phycol. **26**: 299–309.

Chaffin, J. D., T. B. Bridgeman, and D. L. Bade. 2013. Nitrogen constrains the growth of late summer cyanobacterial blooms in Lake Erie. Adv. Microbiol. **03**: 16–26. doi:10.4236/aim.2013.36A003

Chaffin, J. D., S. Mishra, D. D. Kane, and others. 2019. Cyanobacterial blooms in the central basin of Lake Erie: Potentials for cyanotoxins and environmental drivers. J. Gt. Lakes Res. **45**: 277–289. doi:10.1016/j.jglr.2018.12.006

Dekas, A. E., A. E. Parada, X. Mayali, J. A. Fuhrman, J. Wollard, P. K. Weber, and J. Pett-Ridge. 2019. Characterizing Chemoautotrophy and Heterotrophy in Marine Archaea and Bacteria With Single-Cell Multi-isotope NanoSIP. Front. Microbiol. **10**: 2682. doi:10.3389/fmicb.2019.02682

Popa, R., P. K. Weber, J. Pett-Ridge, J. A. Finzi, S. J. Fallon, I. D. Hutcheon, K. H. Nealson, and D. G. Capone. 2007. Carbon and nitrogen fixation and metabolite exchange in and between individual cells of Anabaena oscillarioides. ISME J. **1**: 354–360. doi:10.1038/ismej.2007.44

Steffen, M. M., B. S. Belisle, S. B. Watson, G. L. Boyer, and S. W. Wilhelm. 2014. Status, causes and controls of cyanobacterial blooms in Lake Erie. J Gt. Lakes Res **40**: 215–225.
